# Spike S1 domain interactome in non-pulmonary systems: A role beyond the receptor recognition

**DOI:** 10.3389/fmolb.2022.975570

**Published:** 2022-09-26

**Authors:** Ilaria Iacobucci, Vittoria Monaco, Luisa Canè, Francesca Bibbò, Valentina Cioffi, Flora Cozzolino, Alfredo Guarino, Massimo Zollo, Maria Monti

**Affiliations:** ^1^ Department of Chemical Sciences, University of Naples “Federico II”, Naples, Italy; ^2^ CEINGE Advanced Biotechnologies, Naples, Italy; ^3^ Department of Translational Medical Sciences, University of Naples “Federico II”, Naples, Italy; ^4^ Department of Molecular Medicine and Medical Biotechnologies (DMMBM), University of Naples “Federico II”, Naples, Italy; ^5^ Department of Translational Medical Science, Section of Pediatrics, University of Naples “Federico II”, Naples, Italy

**Keywords:** SARS-CoV-2, proteomics, interactomics, host-virus interaction, COVID-19, spike protein, AP-MS, S1 domain

## Abstract

Severe acute respiratory syndrome coronavirus 2 (SARS-CoV-2) causes Coronavirus Disease 2019 (COVID-19), which, since 2019 in China, has rapidly become a worldwide pandemic. The aggressiveness and global spread were enhanced by the many SARS-CoV-2 variants that have been isolated up to now. These mutations affect mostly the viral glycoprotein Spike (S), the capsid protein mainly involved in the early stages of viral entry processes, through the recognition of specific receptors on the host cell surface. In particular, the subunit S1 of the Spike glycoprotein contains the Receptor Binding Domain (RBD) and it is responsible for the interaction with the angiotensin-converting enzyme 2 (ACE2). Although ACE2 is the primary Spike host receptor currently studied, it has been demonstrated that SARS-CoV-2 is also able to infect cells expressing low levels of ACE2, indicating that the virus may have alternative receptors on the host cells. The identification of the alternative receptors can better elucidate the pathogenicity and the tropism of SARS-CoV-2. Therefore, we investigated the Spike S1 interactomes, starting from host membrane proteins of non-pulmonary cell lines, such as human kidney (HK-2), normal colon (NCM460D), and colorectal adenocarcinoma (Caco-2). We employed an affinity purification-mass spectrometry (AP-MS) to pull down, from the membrane protein extracts of all cell lines, the protein partners of the recombinant form of the Spike S1 domain. The purified interactors were identified by a shotgun proteomics approach. The lists of S1 potential interacting proteins were then clusterized according to cellular localization, biological processes, and pathways, highlighting new possible S1 intracellular functions, crucial not only for the entrance mechanisms but also for viral replication and propagation processes.

## Introduction

The pandemic of the novel severe acute respiratory syndrome coronavirus 2 (SARS-CoV-2), which emerged in the last months of 2019 in China, is still posing significant threats worldwide. In addition, many SARS-CoV-2 variants (Durmaz et al., 2020) have been isolated, complicating the scenario. The most significant part of the mutations gathers within the capsid glycoprotein Spike (S), which is the main responsible for the virus attachment through the recognition and the binding of one of the well-known host cell receptors, the angiotensin-converting enzyme 2 (ACE2) (Shang et al., 2020). Especially at the beginning of the pandemic, the most extensive parts of the information on SARS-CoV-2 have been retrieved by comparison with other Coronaviruses, and/or other viruses. ACE2 itself was initially investigated because it was known to be responsible for SARS-CoV recognition and entry into human cells (Turner et al., 2004). The interaction Spike-ACE2 has been well described: the first step of recognition has been reported to be played by the S1 domain of the glycoprotein, while the S2 and S2’ subunits, generated following Spike proteolytic activation by human proteases (e.g., furin) (Chambers et al., 2020), locally promote host membrane destabilization favoring viral entry. However, it cannot be ruled out that, besides the main role exerted by the glycoprotein in the early stage of infection, S may participate in other intracellular functions.

Although ACE2 expression levels are quite low in the lung and the upper respiratory tract (Qi et al., 2020), it remains the primary door for virus access in host cells. However, SARS-CoV-2 has been demonstrated to be able to infect cells not expressing or definitively expressing very low levels of ACE2 (Yang et al., 2020), indicating that the virus may have alternative receptor(s) or co-receptors that participate in the entry mechanism(s). Bioinformatic and computational analyses have also predicted alternative Spike binding proteins based on homology with other viruses (Zhang et al., 2021) or coronavirus host-virus interactions (Perrin-Cocon et al., 2020).

Besides the lungs and upper airways, which are the main targets of SARS-CoV-2 infection, other tissues and/or organs i.e., heart (Azevedo et al., 2021; Bader et al., 2021), brain (Aghagoli et al., 2021), kidney (Bravi et al., 2022; Singh et al., 2022), gut (Villapol, 2020; Lei et al., 2021; Coles et al., 2022, etc) are affected by the virus, with even serious clinical manifestations (Sridhar and Nicholls, 2021). The renal cell line HK-2 and the human colorectal adenocarcinoma cell line Caco-2 have been demonstrated to be infected by SARS-CoV-2 (Yeung et al., 2021; Bojkova et al., 2020) and used as a model in several COVID-19 researches. The identification of alternative receptors expressed in other tissues than the lungs, which anyway are targets of the virus can help in the elucidation of SARS-CoV-2 pathogenicity.

In this paper, we investigated the Spike S1 subunit protein-protein interactions with host membrane proteins derived from the human kidney (HK-2), colon (NCM460D), and colorectal adenocarcinoma (Caco-2) cell lines, by employing an affinity purification-mass spectrometry (AP-MS) approach. To identify possible alternative receptors or to elucidate preliminary events concerning SARS-CoV-2 internalization, the membrane protein extracts have been let to interact with a recombinant form of the Spike S1 domain, by performing a pulldown assay. A shotgun approach was employed for protein identification. The lists of S1 interacting proteins have been deeply analyzed with bioinformatic clusterization tools, thus to further elucidate the molecular mechanisms of SARS-CoV-2 pathogenicity by the recognition of never-described targets, helpful in the development of novel anti-COVID-19 treatments.

## Experimental methods

### Cell culture

The HK-2 and NCM460D cell lines were purchased by the cell culture facility at CEINGE-Advanced Biotechnology. Conversely, Caco-2 cells were seeded onto 6-well plates at a density of 5 × 10^5^ cells per well. Cells were grown in high glucose (4.5 g/L) DMEM (Gibco, Thermo Fisher Scientific, Oxfordshire, United Kingdom) supplemented with fetal calf serum (FBS) (10%; Gibco), non-essential amino acids (1%), penicillin (50 mU/mL), and streptomycin (50 mg/ml). After 18 days (with the medium changed every 48 h), cells were washed twice with cold PBS, collected with scraping, and centrifuged at 3,000 g × 5 min. The supernatants were discarded, and the cell pellets were refrigerated at −80°C until use.

HEK293T-ACE2 cells represent an *in vitro* model to study the mechanisms underlying SARS-CoV-2 infection. These are HEK293T cells stably expressing ACE2 (Angiotensin-converting enzyme 2). Thus, the over-expression of this integral membrane protein allows these cells to be efficiently infected by SARS-CoV-2. The generation of this model is elsewhere described (Ferrucci et al., 2022).

### Pulldown assay

To obtain the membrane protein extracts, the cell lines were treated with Mem-PER™ Plus Membrane Protein Extraction kit (Thermo Fisher Scientific, Waltham, Massachusetts, United States), according to the manufacturer protocol. The Spike S1 recombinant protein was purchased by Abcam (Cambridge, United Kingdom) as a chimera protein fused with a sheep Fc. 2.5 µg of Spike S1 was immobilized on 40 µL of slurry Dynabeads protein G (Thermo Fisher Scientific, Waltham, Massachusetts, United States), and incubated for 2 h at 4°C.

For the pre-clearing step, 1 mg of each membrane extract was previously incubated with naked Dynabeads Protein G (Thermo Fischer Scientific, Waltham, Massachusetts, United States) for 2 h at 4°C to adsorb non-specific proteins. The supernatants were incubated with the resin derivatized with the S1 protein overnight at 4°C. The supernatants were removed, and the resin was washed with membrane extraction buffer provided by the Mem-PER™ kit. The proteins retained in the S1 pulldown and onto the pre-clearing resin were eluted with a buffer containing 5% SDS. The pre-clearing eluates were used as a control.

### Shotgun protocol and mass spectrometry analysis

S1 pulled-down and control eluates from the 3 cell lines were digested with the S-Trap cartridges (Protifi, New York, United States) according to the manufacturer’s protocol. Peptide mixtures were analyzed by nano LC-MS/MS, using the Proxeon nanoEasy II chromatographic system coupled with an LTQ Orbitrap XL mass spectrometer. Peptide mixture fractionations were performed onto a C18 capillary reverse-phase column (200 mm, 75 μm, 5 µm) working at a 250 nL/min flow rate, using a non-linear 5–50% gradient of eluent B (0.2% formic acid, 95% acetonitrile LC-MS Grade) over 260 min. Mass analyses were performed in Data Dependent Acquisition (DDA) mode by fragmenting the 10 most intense ions in the collision-induced dissociation (CID) modality. Protein identification was carried out by MaxQuant software (v.1.5.2.8), using the UniProt Homo Sapiens database as previously described (Palinski et al., 2021). The FDR cutoff for each peptide and protein identification was set up to 0.01. The fixed modification was the carbamidomethyl (Cys), while the variable modifications were Gln- > pyro-Glu (N-term Gln) and Oxidation (Met). Only proteins identified with at least four peptides were considered acceptable. The mass spectrometry proteomics data have been deposited to the ProteomeXchange Consortium via the PRIDE (Perez-Riverol et al., 2022) partner repository with the dataset identifier PXD035709.

### Filtering out AP-MS contaminants

To define the non-specific contaminants identified in the pulldown experiment, we employed the resources from The Resource for Evaluation of Protein Interaction Networks (REPRINT), a collection of computational tools for the analysis of affinity purification mass spectrometry data. First, we queried the Contaminant Repository for Affinity Purification (CRAPome) 2.0 web tool (https://reprint-apms.org) (Mellacheruvu et al., 2013), searching for the human experiment collection. The UniProt codes were inserted in the database interrogating the “Workflow1” and “*H. sapiens*–All” parameters. Contaminants defined as proteins reported in at least 50% of the experiments were discarded from the initial lists. Therefore, we analyzed all sample and control datasets by using the SAINT algorithm to discard from the final list of S1 interactors all proteins present even in a single control, by comparing the intensities as a parameter of identification (Gamboni et al., 2014).

### Bioinformatic analysis

The cell compartment enrichment analysis has been carried out by Funrich 3.1.3 (Fonseka et al., 2021) software by interrogating the Gene Ontology database. The FDR and fold enrichment cutoffs were 0.01 and 3, respectively. The bubble plots have been obtained by GraphPad nine software. The biological processes and pathways over-representation analysis have been done by using the ClueGO 2.5.7 app (Bindea et al., 2009) of the Cytoscape platform. The Bonferroni adjusted *p*-value (FDR) thresholds have been set to 0.001 and 0.01 for biological processes and pathways, respectively.

## Results

### Identification of S1 interactomes in the HK-2, Caco-2, and NCM460D cells

**FIGURE 1:**
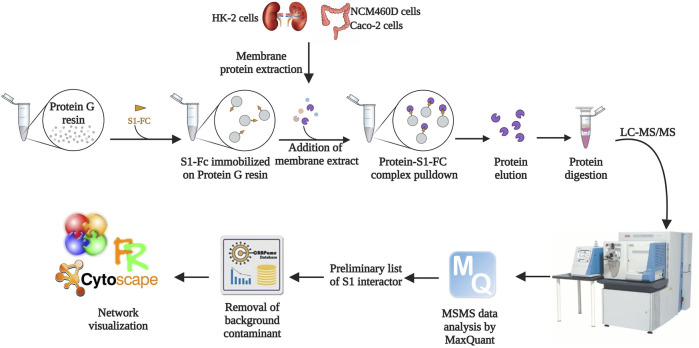
Workflow of the AP-MS approach followed in the present study. The solid support was functionalized with a recombinant form of S1. Membrane protein extracts derived from HK-2, NCM460D, and Caco-2 cell lines were incubated with the resin, and the retained protein complexes were eluted in denaturing conditions. The samples were subjected to a shotgun hydrolysis protocol using trypsin as the proteolytic enzyme. The peptide mixtures were analyzed by nano LC-MS/MS and proteins were identified by the software MaxQuant. The preliminary lists obtained discarding the proteins identified in the negative controls were further filtered with the contaminant database CRAPome. The final lists were analyzed by bioinformatics tools such as Cytoscape.

The early stages of SARS-CoV-2 infection have been investigated by a functional proteomic approach based on AP-MS, for the identification of membrane proteins interacting with S1 subunit from Spike protein in non-pulmonary cell systems, i.e., HK-2, NCM460D, and Caco2 cell lines. The workflow employed is summarized in Figure 1. All cell pellets were lysed to separate cytosolic and integral membrane/membrane-associated protein fractions. The efficiency of the fractionation procedure was monitored by western blot assays, using specific markers for each cell compartment (Supplementary Figure S1). Additionally, also ACE2 was monitored in the membrane extracts of the 3 cell lines. As shown in Supplementary Figure S2A, no signals corresponding to the ACE2 appear in the developments, suggesting the absence or the presence of a very low expression level of this protein in the cell lines under investigation in our conditions. To corroborate this result, we also performed a western blot assay on cytosolic and membrane protein extracts obtained both by wild-type and over-expressing ACE2 HEK293 cells. As shown in Supplementary Figure S2B, a signal corresponding to the ACE2 protein is detectable only in the membrane fraction of the HEK293-ACE2 cell line.

Protein extracts from membranes were incubated onto a solid support conjugates to the S1 domain to pulldown its interacting partners. The membrane protein extracts were solely pre-incubated with the solid supports and the retained proteins were used as control. The proteins eluted from the samples and the controls were identified by a shotgun approach, using MaxQuant software. The protein groups generated for each analysis are reported in Supplementary Table S1. All sample lists were filtered by using computational tools available in the Resource for Evaluation of Protein Interaction Networks (REPRINT). As first, by using the SAINT algorithm, we compared all proteins occurring in the three sample lists with those present in all three pre-cleaning controls: only proteins never occurring in any control were considered for the following steps. This first list including filtered potential S1 interactors from all cell lines was then further filtered out using the Contaminant Repository for Affinity Purification (CRAPome) 2.0 (Mellacheruvu et al., 2013) to discard the most common contaminants for each specific experimental set up. Finally, at the end of this stringent skimming, we obtained 55 interactors in HK-2, 80 in NCM460D, and 85 in Caco-2 cells (Supplementary Table S2). The unexpected identification of a large number of interactors from each cell line suggests the involvement of S1 in much more cell processes rather than the mere recognition of surface receptor(s).

### Cellular components enrichment analysis of S1 interactors

**FIGURE 2:**
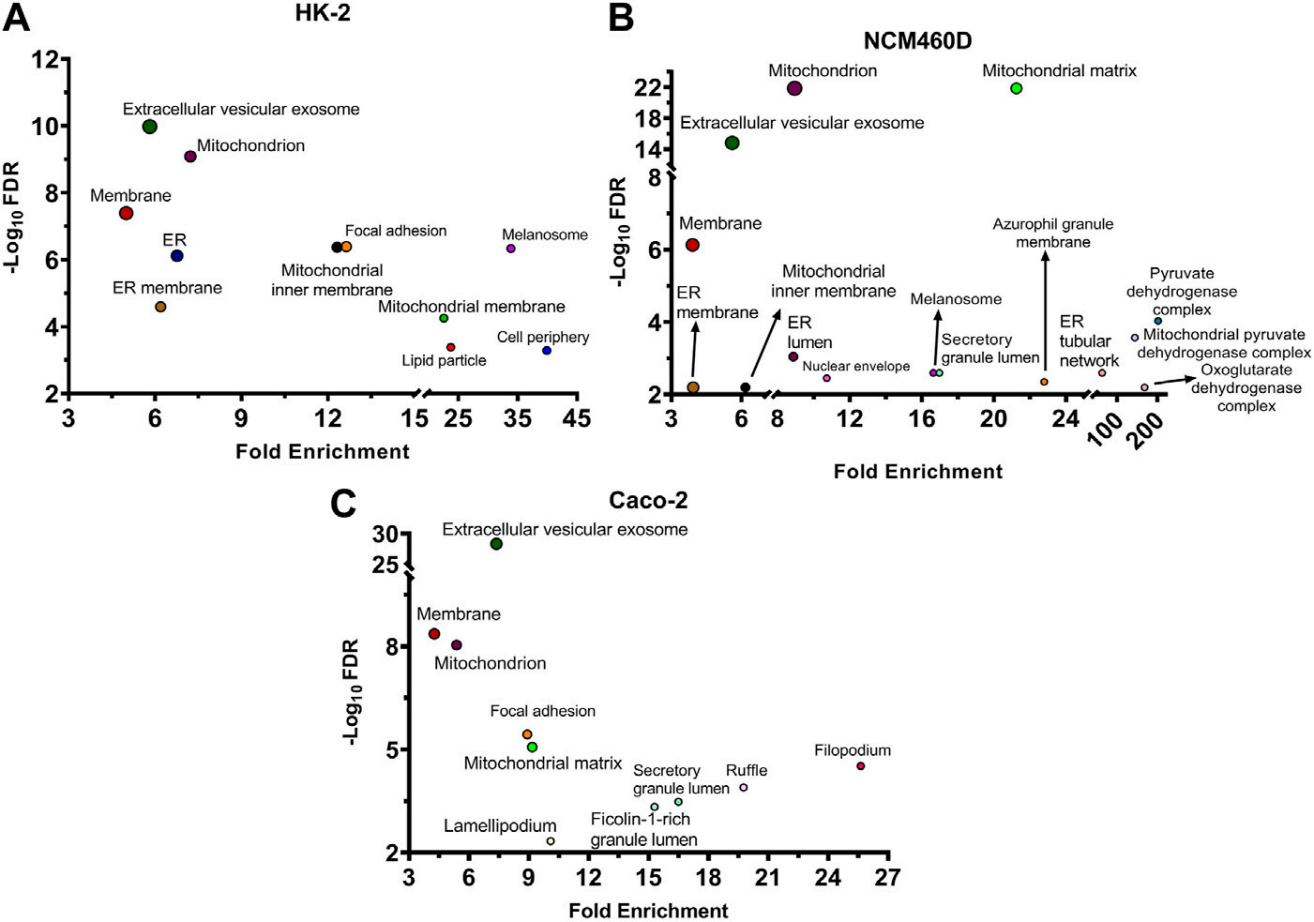
Cellular components analysis of all identified S1 interactors based on Gene Ontology database. For each enriched term, the bubble plot displays the -Log10 FDR versus the fold enrichment value for HK-2 **(A)**, NCM460D **(B)**, and Caco-2 **(C)** cell lines. FDR: False Discovery Rate.

To define cell components enriched in the interactomes of the S1 subunit of Spike within the three different cell lines, an over-representation analysis has been performed. The proteins identified in all cell systems were functionally analyzed by FunRich 3.1.3, interrogating the Gene Ontology database to reveal the enriched subcellular locations. The terms were considered significant when the *p*-value adjusted with Benjamini–Hochberg (FDR) was <0.01 and the fold enrichment was higher than 3. The results are shown in the bubble plot in Figure 2, reporting the -Log_10_FDR vs. fold enrichment for each term.

As expected, one of the most significantly enriched terms found in all cell lines concerned membrane-related proteins, although other categories were significantly represented. In all the datasets the “extracellular vesicle exosomes” category obtained the highest score in terms of FDR, suggesting a possible role of this Spike domain also in the exit of viral particles. Indeed, it is well known that extracellular exosomes are membrane-budding vesicles used by viruses to cloak their viral genome and entry and spread into uninfected host cells (Ju et al., 2021). The exosomes have been recently earning interest for their emergent roles also in SARS-CoV-2 infection mechanisms (Barberis et al., 2021; Hassanpour et al., 2020). In fact, the virions cloak the genome in these membrane-budding vesicles (Jia et al., 2021) and hijack the host immune system because the “membrane wrapping” protects the virus from neutralizing antibodies (Borowiec et al., 2021; Jia et al., 2021). Moreover, the Spike protein has been demonstrated to manipulate the cargo sorting of host exosomal vesicles delivering pro-inflammatory molecules (Mishra and Banerjea, 2021). In light of that, our results confirm a possible physical affinity of S1 towards such exosome component proteins, suggesting a role of Spike also in the spread stage of infection.

The renal cell line HK-2, as well as NCM460D datasets, displayed also a more pronounced presence of the mitochondrial compartment, as hinted by the general term “mitochondrion” with fold enrichments of about seven in both datasets, although the category “mitochondrial matrix” is more enriched in NCM460D (Figure 2B) while “mitochondrial inner membrane” is peculiar of HK-2 (Figure 2A).

Similarly, in these 2 cell lines, another category strongly enriched refers to the endoplasmic reticulum (ER). It is well known that once entered into the host cells, the viral particles traffic towards lysosomes, where disassemble to release their genomic RNA that starts to be translated by host molecular machinery (Ghosh et al., 2020). Since many viral proteins, mainly those associated with the envelope, carry specific post-translational modifications (PTMs), such as glycosylation, they have to be translated into ER, where the assembly process of viral particles starts (Fukushi et al., 2012). The direct or indirect interactions between S1 and several ER proteins may suggest that the complex processes occurring in this cell compartment and important for the viral life cycle might physically involve Spike protein.

In addition to the above-mentioned main categories, the presence of other cell structural units (i.e., focal adhesion, lamellipodium, ruffle, membrane raft) suggests a role of S1 in the interaction with the actin cytoskeleton dynamics at the plasma membrane as well as the interaction with structure binding extracellular matrix (ECM) (Figure 2C). All these peripheral cell processes are well known to have a pivotal role in virus propagation mechanisms into host cells (Kloc et al., 2022) and are much more representative in Caco-2, than in the other cell lines under investigation.

### Comparison of three interactomes protein components

To make hypotheses about the presence of potential alternative or cooperative cell surface receptors in the viral entrance processes, we specifically focused on proteins strictly associated with membranes. By using the Uniprot “Retrieve/ID Mapping” function (Pundir et al., 2016), all datasets were further screened according to the Gene Ontology cellular component category, and only those genes, whose annotation contained the term “membrane” or “cell surface”, were extracted by the total lists and subjected to further bioinformatic analyses. In particular, among all the potential S1 interactors 48, 54, and 50 proteins were flagged as membrane proteins (highlighted in pink within the single cell line protein list in Supplementary Table S2) for HK-2, NCM460D, and Caco-2 samples, respectively.

**FIGURE 3:**
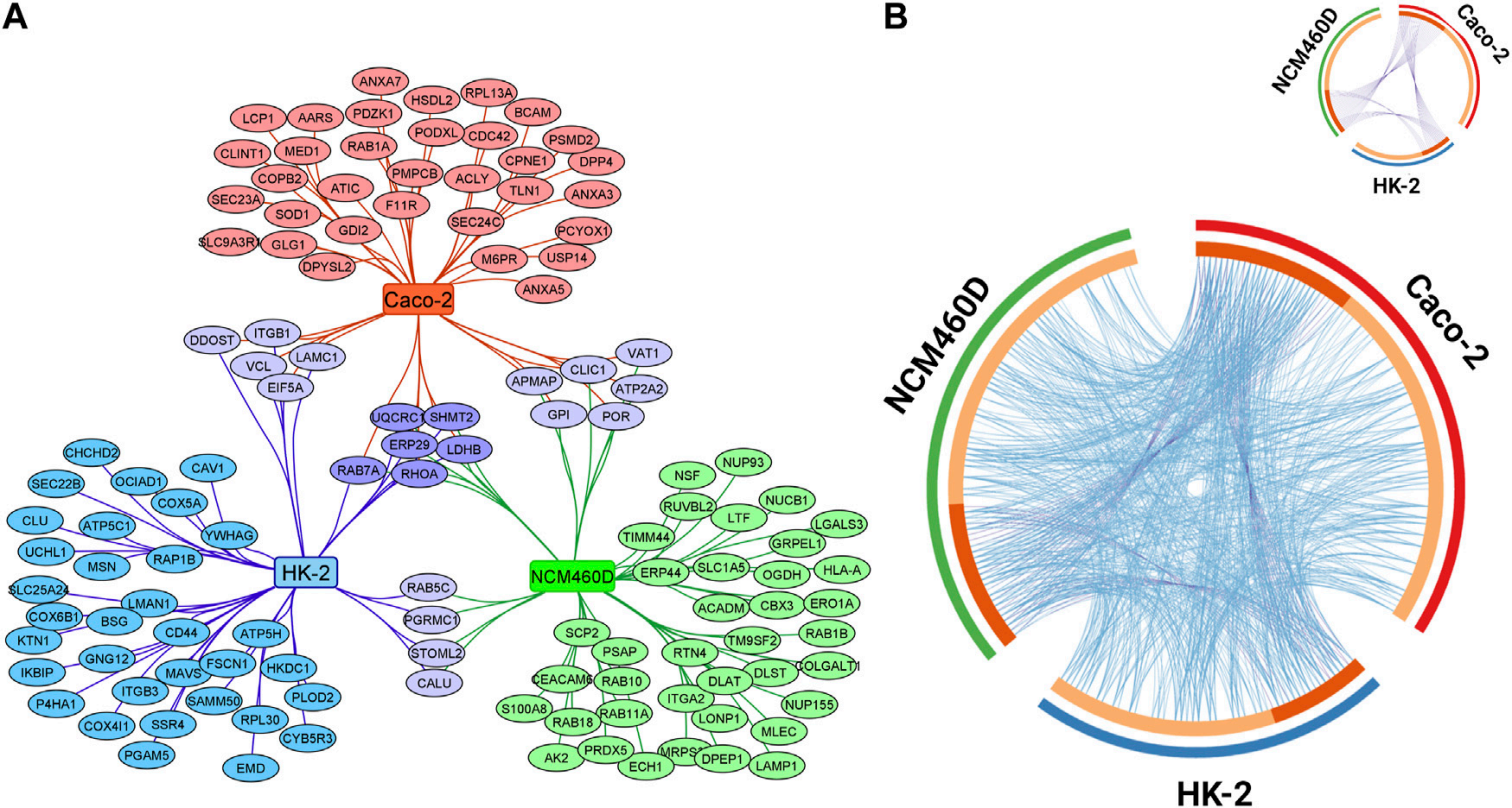
Visualization of the datasets obtained by S1 pulldown in HK-2 NCM460D, and Caco-2 cells. **(A)** Genes specific for HK-2, NCM460D, and Caco-2 are depicted in the graph as blue, green, and red nodes, respectively. The shared proteins are reported as violet circles. **(B)** Circos plot representing the overlap between gene lists at the gene and GO level is reported. Purple curves link identical genes and are represented in the insert figure. The inner circle represents gene lists, where hits are arranged along the arc. Genes that hit multiple lists are colored in dark orange, and genes unique for a list are shown in light orange. Blue lines link genes that belong to the same enriched ontology term.

These three lists were compared, and the overlapping proteins among all the subsets were visualized with Cytoscape as reported in Figure 3A. Overall, in comparison to the total number of proteins identified in each dataset, only six proteins (*LDHB*, *UQCRC1*, *ERP29*, *RHOA*, *SHMT2*, *RAB7A*) were shared among the three gene lists and an analogous number was separately shared at least between two datasets. This finding suggests that the S1 interactomes are definitively cell-specific if we look at single-gene identity.

Among the proteins shared by all datasets, *RAB7A* is a small GTPase that cycles between active GTP-bound and inactive GDP-bound states. In its active state, *RAB7A* binds to a variety of effector proteins playing a key role in the regulation of endo-lysosomal trafficking. It has been already described to be involved in the early stage of SARS-CoV-2 infection in human alveolar epithelial cells (Daniloski et al., 2021). Analogously to *RAB7A* also *RhoA* is a small GTPase that, in its active state, binds to a variety of effector proteins involved in the regulation of many cellular processes, such as cytoskeletal dynamics, cell migration, and cell cycle. It affects also the signal transduction pathway starting from several plasma membrane receptors for the assembly of focal adhesions and actin stress fibers (Quilliam et al., 1996; Vincent and Settleman, 1997; Vabres et al., 2019). *RhoA* has been already found as an S1 interactor within endothelial cells in the brain (DeOre et al., 2021) and it is described as a key mediator in the disruption of the brain-blood barrier by its Spike-induced activation. The recurrent presence of these GTPase proteins in so different cell lines as Spike interactors, suggests for *RAB7A* and *RhoA* a general crucial role in the SARS-CoV-2 propagation process.

*UQCRC1* and *SHMT2* are mitochondrial proteins: the first is a component of the ubiquinol-cytochrome c oxidoreductase, localized within the inner mitochondrial membrane; the second is an enzyme that catalyzes the cleavage of serine to glycine accompanied by the production of 5,10-methylenetetrahydrofolate, an essential intermediate for purine biosynthesis (Zheng et al., 2013) (Morscher et al., 2018) (Giardina et al., 2015) (García-Cazorla et al., 2020). So far, no data on their specific role in COVID-19 pathology has been reported.

Differently, lactate dehydrogenase B (*LDHB*), an enzyme involved in pyruvate anaerobic metabolism, has been largely described as altered in infected cells (Mullen et al., 2021) (Tang et al., 2021). Although it is mainly localized in the cytosol, its presence on cell and exosome surfaces has been also detected (Li et al., 2012).

*ERP29* is a PDI-like protein, mainly resident in the ER. It has been already described in polyomavirus infection as the protein able to trigger the penetration of the ER membrane by altering the conformation of coat protein VP1, enabling the virus to interact with membranes (Rainey-Barger et al., 2009) (Rainey-Barger et al., 2007). This interaction between Spike S1 and *ERP29* might be subsequent to the SARS-CoV-2 entrance but functional to the early stage of the viral exocytic pathway, which begins with vesicle budding from the ER.

Despite the poor sharing of genes among the three conditions, we moved to evaluate their functional overlapping according to the GO terms. The results are graphically reported in the Circos plot in Figure 3B (Zhou et al., 2019). The light orange arcs represent the gene lists from each cell line; the dark orange portions, roughly corresponding to one-third of each list, includes genes shared at least between two datasets and connected by purple curves. Differently, the light blue curves, linking the genes belonging to the same enriched ontology term, revealed a large and homogeneous distribution among all gene lists, demonstrating a high functional overlapping degree in all membrane annotated S1 interactomes.

As reported, the comparative analyses of the three interactome components apparently do not reveal the presence of novel Spike receptors among the few shared proteins, although all the latter are known to play different roles in viral processes. However, GO analyses summarized in the circos plot let to hint that the S1 subunit may be involved in a large number of shared cell processes, although contacting different partners in dependence on the specific cell type. The dense network of functional connections highlighted in the cycle plot further confirms the hypothesis that S1 and, therefore the entire Spike protein, might play additional crucial roles in different stages of the viral life cycle.

### Biological processes and pathways enriched in S1 membrane interactomes

**FIGURE 4:**
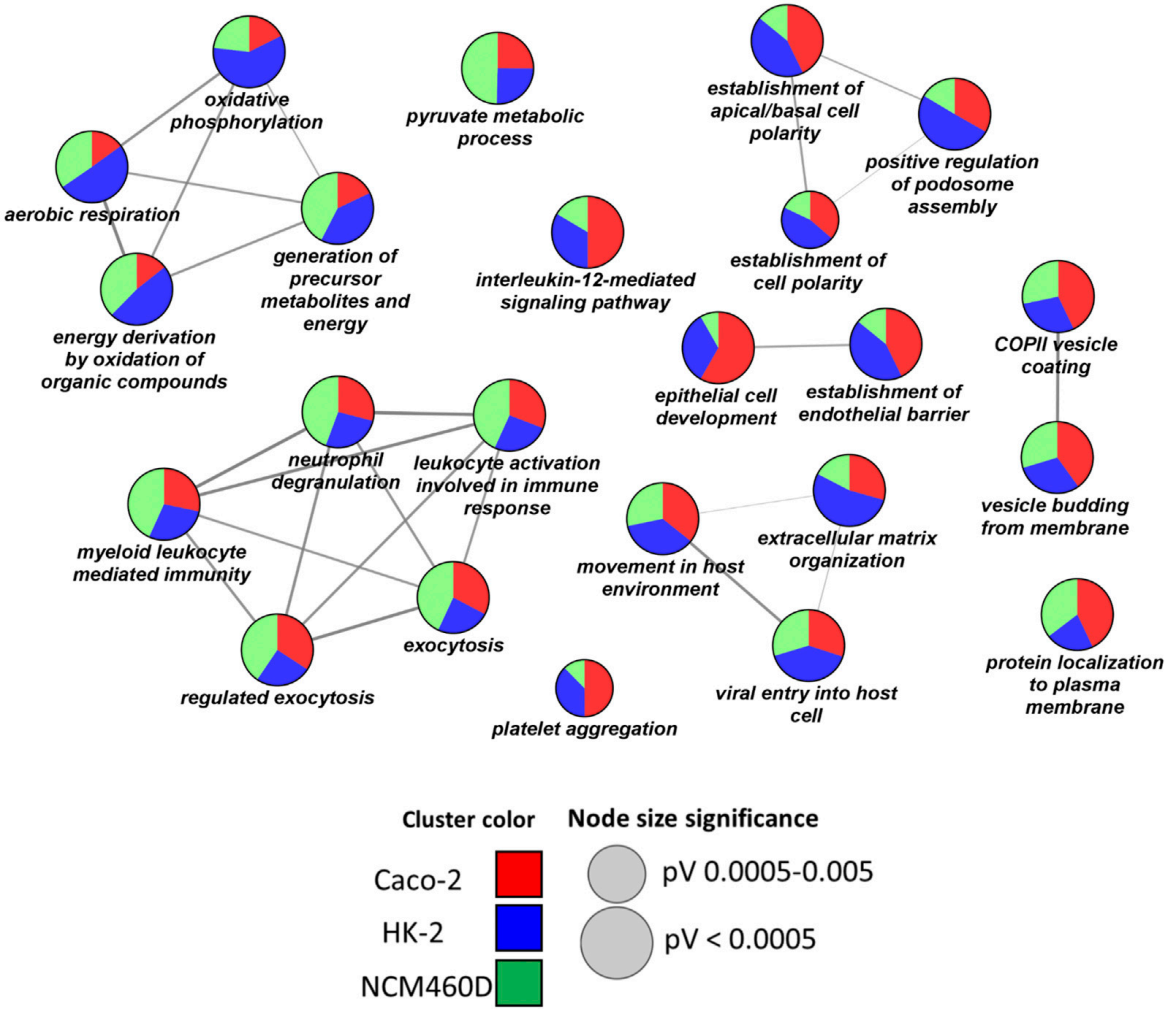
Biological processes enrichment analysis by ClueGO. Network representation of the enriched processes in each cell line. The terms enriched in HK-2, NCM460D, and Caco-2 are represented as blue, green, and red pie slices, respectively. The slice extent represents the contribution of the specific dataset to the term. The node sizes are proportional to the FDR values.

Prompted by these findings, we decided to deeply investigate the biological processes over-represented in our datasets. In detail, a functional clusterization has been performed by the ClueGO app (version 2.5.7) provided by the Cytoscape platform, to highlight the nature of the common processes shared by all the datasets (FDR<0.001, Figure 4). We fixed 60% as the specificity threshold: in other words, the terms including more than 60% of genes from one cluster are considered peculiar to that cluster. In Supplementary Table S3, the complete analysis output is reported. The red, green, and blue clusters have been derived from the Caco-2, NCM460D, and HK-2 datasets, respectively. The pie slices for each process refer to the percentage contribution of each dataset to the term.

As shown in Figure 4, no functional cluster is exclusively assigned to a single dataset. Differently, each cell line shared in different percentages all the statistically significant terms (FDR<0.001). Among the enriched processes we found “viral entry into the host cell”, “extracellular matrix organization”, “movement in the host environment”, and “vesicle budding from membrane”. Altogether these processes describe the different complex steps of the viral entry process into the host cells, starting from the receptor recognition to the viral genome release in the cytosol. Starting from the analysis of potential molecules involved in the recognition phase (“viral entry into the host cell” term, Table 1), some proteins such as *CD147*/Basigin (found in HK-2) and *DPP4* (found in Caco-2) have already been proposed as alternative receptors to ACE2 in SARS-CoV-2 infection (Wang et al., 2020a) (Li et al., 2020).

**TABLE 1:** List of receptors clustered in the “viral entry into the host cell” term. For each protein the gene name, the membership dataset, the viruses, and the literature references in which the protein has been demonstrated to be a receptor.

	Dataset				
Gene name	NCM460D	HK-2	Caco-2	Virus	References
*BSG*		Yes		SARS-CoV-2, HCMV, measles virus	(Vanarsdall et al., 2018)*,* (Wang et al., 2020a)*,* (Watanabe et al., 2010)
*DPP4*			Yes	SARS-CoV-2 (*predicted*), MERS-CoV	Li et al. (2020)
*ITGB1*		Yes	Yes	Human echoviruses 1 and 8, Human rotavirus A	(Barillari et al., 1999)*,* (Bergelson et al., 1993)*,* (Arias et al., 2002)
*ITGB3*		Yes		KSHV, HCMV, hMPV, HIV-1	(Othman et al., 2022)*,* (Garrigues et al., 2008)*,* (Wang et al., 2005)*,* (Wei et al., 2014)
*ITGA2*	Yes			Human echoviruses 1 and 8, Human rotavirus A	Barillari et al. (1999)
*LAMP1*	Yes			Lassa virus	Jae et al. (2014)
*SLC1A5*	Yes			Several retroviruses	Rasko et al. (1999)
*F11R*			Yes	Human Rotavirus strain WA	Torres-Flores et al. (2015)

In particular, Wang et al. demonstrated the direct *in vitro* interaction between the transmembrane glycoprotein *CD147* and the receptor-binding domain (RBD) of Spike by Surface Plasmon Resonance (SPR) and Enzyme-Linked Immunosorbent Assay (ELISA). The binding between *CD147* and Spike was also demonstrated *ex vivo* by Co-IP assays and their co-localization was assessed *in vivo* in kidney tissues by immune-electron microscope analysis. They also revealed an endocytic process of SARS-CoV-2 mediated by *CD147*, demonstrating the co-localization of Spike and *CD147* with Rab5, a key regulator of the endocytic pathway (Saitoh et al., 2017), also identified either in our NCM460D and HK-2 datasets. On the other hand, also the dipeptidyl peptidase 4 (*DPP4/CD26*), previously recognized as the receptor for the *Middle-East Respiratory Coronavirus* (MERS-CoV) (Du et al., 2017) (Raj et al., 2013) has been predicted as a potential Spike interactor, according to its high affinity for the RBD domain of SARS-CoV-2, as inferred by docking calculations (Li et al., 2020).

Moreover, the presence of integrins *ITGB1* (in Caco-2 and HK-2), *ITGB3* (in HK-2), and *ITGA2* (in NCM460D), belonging to a family of integral plasma membrane proteins, suggested a common role for these proteins in the virus-host interaction in all cell lines. In fact, it is well known that, despite other Coronaviridae*,* the Spike S1 domain from SARS-CoV-2 uniquely contains the RGD (Arg-Gly-Asp) sequence, a motif strongly conserved in all integrins protein ligands (Ludwig et al., 2021). This motif has been proposed as the attachment site for integrins so far (Makowski et al., 2021; Sigrist et al., 2020; Simons et al., 2021), although, recently, also interactions RGD-independent between Spike and other integrins have been suggested (Beaudoin et al., 2021; Othman et al., 2022).

Supporting the relevant roles of this protein family in viral recognition of target cells, the *ITGB3* integrin has been already identified as responsible for the internalization process of several other viruses, such as Kaposi’s sarcoma-associated herpesvirus (KSHV) (Garrigues et al., 2008), human cytomegalovirus (HCMV) (Wang et al., 2005), human metapneumovirus (hMPV) (Wei et al., 2014), human immunodeficiency virus type-1 (HIV-1) (Barillari et al., 1999). Moreover, the heterodimer *ITGA2*/*ITGB1* has already been described as a receptor for human echoviruses one and 8 (Bergelson et al., 1993) and human rotavirus A (Arias et al., 2002; Graham et al., 2003).

Among the remaining receptors clustered in the “viral entry into the host cell” term, we found *LAMP1,* and *SLC1A5* in NCM460D, and *F11R* in Caco-2 cells.

*LAMP1* has been reported as the receptor for the Lassa virus (Jae et al., 2014), *SLC1A5* for many retroviruses (Rasko et al., 1999), and *F11R* is the receptor for human rotavirus strain WA (Torres-Flores et al., 2015).

Following the recognition phase, the virus endocytosis, accompanied by the endocytic vesicle formation, starts from the actin and cytoskeleton remodeling (Wang et al., 2021), as suggested by all the proteins grouped in the “extracellular matrix organization” and “movement in the host environment ontologies, including *FSCN1* (in HK-2), *TLN,* and *LCP1* (in Caco-2), *VCL* (in HK-2 and Caco-2). In this context, we also identified *CD44* protein in the HK-2 dataset, a cell-surface protein, able to promote actin-mediated cytoskeleton reorganization through its binding to Rho GTPases like *RHOA* (Bourguignon et al., 2004), which is shared by all the datasets as reported in Figure 3A. Similarly, the *S100A8* protein belonging to the calprotectin complex, known to affect cytoskeleton rearrangement (Wang et al., 2018), has been reported to promote the entry of pentamer-expressing human cytomegalovirus (HCMV) into epithelial and endothelial cells (Vanarsdall et al., 2018). Surprisingly, it has been found upregulated in fatal cases of SARS-CoV-2 (Wu et al., 2020).

Finally, the identification of *CDC42*, Caveolin-1 (*CAV1*), and *CLINT1* within these three interactomes of S1, suggests the occurrence of SARS-CoV-2 internalization through different endocytosis mechanisms (Chi et al., 2013) (Nomura, 2005) (McPherson and Ritter, 2005).

The RABS family is also highly represented within several Biological Processes, such as “movement in the host environment” and “exocytosis”. In particular, *RAB7A* and *RAB5C*, the first identified in all datasets and the latter both in HK-2 and NCM460D interactomes, are master genes in the viral particles endocytic route towards the lysosome. Danilosky et al. showed that *RAB7A* is essential for SARS-CoV-2 initial attachment and endocytosis; moreover, the loss of *RAB7A* reduces viral entry by sequestering ACE2 receptors inside cells through altered endosomal trafficking (Daniloski et al., 2021). On the other side, the role of *RAB5C* in viral infections and cellular immune responses remains poorly understood, although it has been demonstrated that numerous viruses such as influenza virus (IV), Semliki Forest virus, adenovirus (AdV), vesicular stomatitis virus, and Japanese encephalitis virus, require its presence for virus survival (Wang et al., 2020b). The occurrence also of *LAMP1*, *S100A8*, *PSAP*, and *M6RP* can take into account the endosomal vesicle maturation into lysosomes needed for the pathogen’s life cycle into the host cells (Zhou et al., 2015). The presence of these lysosomal proteins suggests additional crucial roles exerted by Spike during the journey of the viral particles within the host cell towards the lysosomes, maybe acting as an anchor necessary to ensure the stable transport of the virus through the various sub-cellular compartments.

To further delineate the additional roles of Spike S1 in the investigated cell lines, a pathways over-representation analysis by the ClueGO platform interrogating the Reactome database was carried out (see Supplementary Table S4 for the complete output) (Jassal et al., 2020).

**FIGURE 5:**
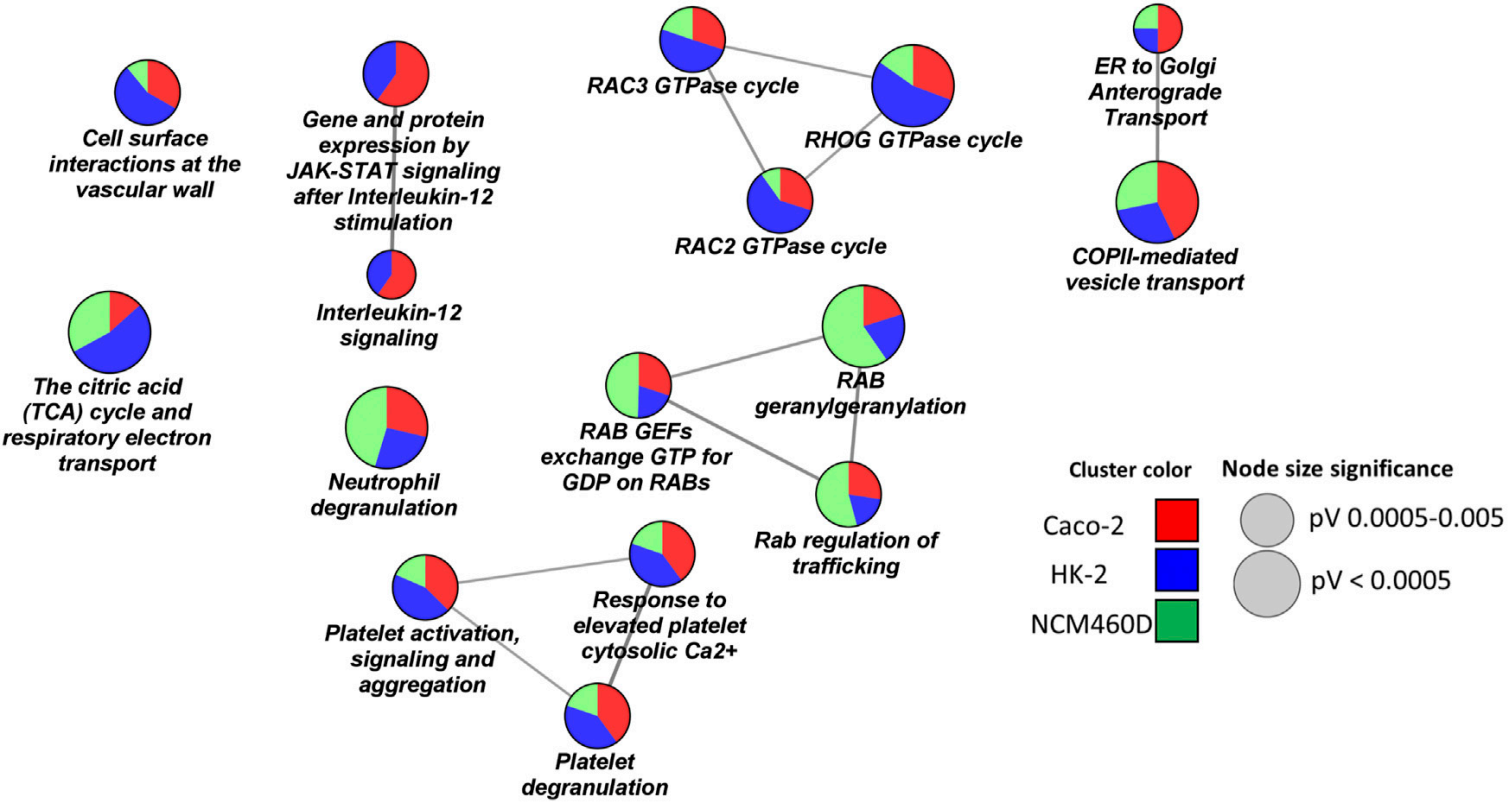
Pathways enrichment analysis by ClueGO based on Reactome database. Networks representation of the enriched pathways in each cell line. The terms enriched in HK-2, NCM460D, and Caco-2 are represented as blue, green, and red pie slices, respectively. The slice extent represents the contribution of the specific dataset to the term. The node sizes are proportional to the FDR values.

In Figure 5 clusters of interesting pathways were detected. As already reported for biological processes, the red, green, and blue clusters have been derived from the Caco-2, NCM460D, and HK-2 datasets, respectively. The pie slices for each process refer to the percentage contribution of each dataset to the term.

The presence of pathway terms related to traffic processes (e.g., *Rab* regulation of trafficking, ER to Golgi anterograde transport) suggests a role for Spike, both in viral particles endocytosis and exocytosis. The interactions of specific envelope proteins with host receptor proteins activating endocytosis pathways have been already discussed above.

Furthermore, the proteomics experiments identified several proteins belonging to the intermediate compartment (IC), such as *ERGIC-53* and several Rabs proteins, which are involved in the anterograde trafficking towards the plasma membrane. The role of their interaction with Spike could be critical for the correct viral assembly process since, for coronaviruses (CoVs), it has been reported that it occurs by budding into the lumen of the intermediate compartment (IC) at the interface between the endoplasmic reticulum (ER) and Golgi stacks (Hartenian et al., 2020).

*ERGIC-53*/*LMAN1* is a cargo receptor required for glycoprotein trafficking at the early steps of the exocytic route towards the PM. This protein has already been described to assist the traffic of newly synthesized viral particles in arenavirus, hantavirus, coronavirus, orthomyxovirus, and filovirus glycoproteins (GPs) (Klaus et al., 2013). It has been demonstrated that the loss of *ERGIC-53* or its activity leads to a defective assembly of virions that are unable to attach host cells. Interestingly, *ERGIC-53* specifically interacts with class I viral fusion glycoproteins which also include the SARS-CoV-2 Spike protein (Pasquato and Kunz, 2013). In the NCM460D cell line, much more than in the others, we assist to a high enrichment in Rabs proteins involved in the exocytic pathway such as *RAB1A* and *RAB1B*. In the absence of *RAB1A/B*, the viral glycoproteins have been demonstrated to be unable to traffic from the ER to the assembly compartment (Zenner et al., 2011). GTPase *RAB11A*, in particular, regulates endocytic recycling, a direct route from IC to the plasma membrane. Indeed, the intermediate compartment (IC) not only is known to be a linker between the ER and the Golgi compartments but is also implicated in Golgi-independent transport routes to the plasma membrane. This alternative exocytic process has been described to increase the yield and the release of active viral particles (Prydz and Saraste, 2022).

As already suggested by biological processes, also the pathways analysis highlights a role of the Spike subunit in affecting mitochondrial functions, confirming a possible interplay between the Spike S1 domain and the energetic cell metabolism in almost all analyzed cell lines, with a more marked incidence in the HK-2 renal cells (Figure 5). Indeed, in HK-2, more than in the other 2 cell lines, the SARS-CoV-2 Spike S1 subunit interactome was enriched in mitochondrial enzymes of the TCA cycle (e.g., *OGDH*, *DLAT*, and *DLST*), and several subunits of the electron transport chain belonging to the ubiquinol-cytochrome C oxidoreductase (e.g., *UQCRC1*) or to cytochrome C oxidase (*COX4I1*, *COX5A*, *COX6B1*) complexes and from ATPase (e.g. *ATP5C1*, *ATP5H*). These data are coherent with recent findings in which several authors demonstrated metabolic reprogramming also at the level of both TCA (Barberis et al., 2020) (Liu et al., 2022) and oxidative phosphorylation (Santos et al., 2021), leading to an impairment of ATP synthesis and the contemporary activation of anaerobic metabolic pathways induced by SARS-CoV-2 infection (Jamison et al., 2022). To the best of our knowledge, for the first time, our data suggest a possible role of the Spike S1 subunit in physically mediating these phenomena.

## Discussion

Severe acute respiratory syndrome coronavirus 2 (SARS-CoV-2) is a viral strain belonging to the Coronaviridae family and the *Betacoronavirus* genera. Coronavirus virion contains four main structural proteins: nucleocapsid protein (N), transmembrane protein (M), envelope protein (E), Spike protein (S), and 15–16 non-structural proteins (Gildenhuys, 2020). Of particular interest is the S protein which “decorates” the surface of the virus by forming characteristic protuberances, making it looks like a crown. During the entrance process, the S protein is proteolytically processed in two subunits: S1 and S2. The first subunit is mainly involved in the recognition and interaction of viral particles with surface host receptors such as ACE2; the latter mediates the fusion of the viral capsid with the host cell membrane. Although COVID-19 mainly affects the respiratory tract, other organs have been identified as further SARS-CoV-2 targets, such as the kidneys and the gastrointestinal tract, leading to severe dysfunctions and damages (Torjesen, 2021; Hunt et al., 2021). Indeed, both renal HK-2 and the adenocarcinoma colorectal Caco-2 cell lines have been reported to be infected by SARS-CoV-2 (Yeung et al., 2021; Shuai et al., 2020), with inflammatory response mainly mediated by the S protein interaction with the cell surface, such as demonstrated in Caco-2 cell lines in terms of NFk-beta activation and *CXCL10* secretion (Poeta et al., 2021).

In this work, by applying an AP-MS workflow (Iacobucci et al., 2021), we looked both for possible Spike additional human receptors and its potential involvement in other intracellular processes in non-pulmonary systems. To this aim, we focused on the recognition domain of Spike protein, S1, to perform a pulldown assay using the membrane protein extracts from the gut (i.e., NCM460D, Caco-2) and renal (HK-2) cell lines, to isolate S1 host interacting partners and protein complexes involving it. Shotgun proteomics approaches were used for protein identification and several bioinformatic platforms for functional data analysis. Despite the relatively low number of totally shared genes, the analysis revealed a general convergence of almost all interactors towards a few common ontology terms.

All types of functional analyses carried out to correlate the various interactomes to cellular components, biological processes or pathways have highlighted the correlation of S1 with multiple processes that go beyond the receptor recognition phase, and include a series of steps critical for the entire virus life cycle, from its internalization to the stages of virion propagation. In particular, the virus life cycle is a very complex multi-step process that initiates with the viral attachment to host cells and carries on through endocytic intracellular trafficking, genome replication, translation, and assembly of novel viral particles, and their exocytic releasing. To carry out these processes, many host proteins localized in the endo-lysosomal system, endoplasmic reticulum (ER), and Golgi apparatus must be recruited to work in a concerted way.

**FIGURE 6:**
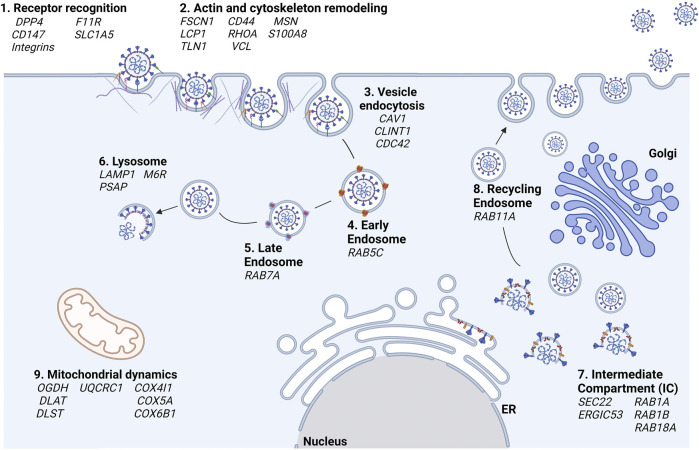
Hypotheses of the cellular processes in which S is involved according to proteomics results. In bold black are reported the cellular processes and in italics the associated genes, respectively.

The proteomics experiments allowed us to identify many host protein targets and to ascertain the role of the S1 domain in all phases of the interaction between the pathogen and the three different host cell lines. In Figure 6, according to their functions and cell localization, the identified proteins are distributed along the known pathways in which they are involved. In addition to the already known Spike’s receptors, like *CD147* and *DPP4*, proteins belonging to the integrin family and transferrin receptor were found and proposed as novel S1 targets on the host cell surface (step 1, receptor recognition). The identification of several proteins associated with cytoskeleton remodeling at the plasma membrane, such as *FSCN1, PFN1,* etc. (step. 2, actin and cytoskeleton remodeling) suggests the involvement of S1 in vesicle formation, both using endocytic clathrin- and caveolin-dependent processes (step 3, vesicle endocytosis). Once originated, vesicles move from the plasma membrane and have to be translocated to endo-lysosomal systems, through continuous actin cytoskeleton rearrangements. The endosomal vesicles go to maturation from the early to the late endosomes and finally to lysosomes (steps 4, 5, and 6, early and late endosomes, lysosomes) in which the viral particles disassembly release the genome into the host cell cytosol, where they are translated and replicated by using host molecular machineries. According to our proteomics results, these processes might be all mediated by the interaction of the S1 domain of SARS-CoV-2 and the identified host proteins.

The formation of the novel virions is accomplished at the level of the intermediate compartment (IC), the region between the ER and Golgi compartment, as already reported for other *Coronaviruses* (Saraste and Prydz, 2021) (step 7, Intermediate Compartment (IC)). The traffic of the newly formed viral particles might be carried out through the interaction of Spike with host proteins such as *RAB11A* (step 8, recycling endosomes). The involvement of the Spike protein within ER-Golgi tethering has also been described by JR St-Germain and collaborators who, like us, had found that Spike interacts with *ERGIC53*, *SEC22B* (St-Germain et al., 2020). In the same paper, the interaction of Spike and of other SARS-CoV-2 proteins with many host proteins involved in vesicular trafficking (such as those belonging to the RABs family) was highlighted.

Our results highlighted also an interplay between SARS-CoV-2 S1 Spike’s S1 domain and human proteins associated with the energetic cell metabolism in almost all analyzed cell lines, with a more marked incidence in the HK-2 renal cells (step 9, mitochondrial dynamics). A large number of studies have found that several viral infections could change mitochondrial dynamics, alter their metabolic status, mediate mitochondrial-induced cell death, and evade the host’s innate immune response to ensure their spread and maintain intracellular survival, respectively. Although mitochondria play an important role in the interaction between the host and the virus, other SARS-CoV-2 proteins than S have been already described to affect mitochondria functions (e.g., Orf9b, Nsp8, ORF9c, M) (Singh et al., 2020; Gao et al., 2021).

From an overview of our results also compared to others already published, it is important to highlight that the overlap of host proteins that interact with S1 both in our experimental contexts and in relation to other interactomes already published by other authors is very poor. This observation was already reported by Bamberger et al. (Bamberger et al., 2021). The explanation of this phenomenon could be attributed to the fact that various studies have been carried out in different cell lines, in which, as we have shown, virus-host interaction processes are often common, although the proteins involved are not the same. Furthermore, it is important to underline that, as previously mentioned, most of Spike’s interactomics studies started from the analysis of immunoprecipitates of cells already infected or transfected with vectors expressing the viral protein of interest. In both cases, the focus of the experiment was aimed at monitoring intracellular interactions, losing information on the recognition of extracellular membrane receptors. Differently, our main initial aim was the identification of S1 receptors or co-receptors alternative to ACE2 in non-pulmonary cell systems, and this drove us in the choice of experimental strategy. Future studies are needed to deeply investigate the functional interaction of S with mitochondrial factors to clarify how and if it can regulate host metabolic reprogramming for viral survival.

## Conclusion

The unbiased proteomic approach, employed for the investigation of S1 interactomes in different non-pulmonary cell lines, allowed the identification of many interacting proteins, several cell-specific, but also others shared among the gut and kidney systems. These proteins might constitute novel potential targets for preventive and/or curative pharmacological treatments, not only at the level of the entry phase of the virus into the cell but also in subsequent steps, in the light of the surprising multiple roles of Spike proteins (through its S1 domain) in all fundamental steps for the survival and spread of the infectious agent. Our results suggest that Spike protein has several unexpected roles that go far beyond that of mediator of receptor recognition on host cells.

## Data Availability

The datasets presented in this study can be found in online repositories. The names of the repository/repositories and accession number(s) can be found in the article/Supplementary Material.
