# Supplementary material for: Spike S1 domain interactome in non-pulmonary systems: A role beyond the receptor recognition
Source: Front Mol Biosci. 2022 Sep 26;9:975570. doi: 10.3389/fmolb.2022.975570 (PMC9550266; doi:10.3389/fmolb.2022.975570)
Supplement: Supplementary file 3 [file DataSheet1.PDF]

Supplementary Material

Spike S1 domain interactome in non-pulmonary systems: a role beyond the receptor recognition

Ilaria Iacobucci[1,4], Vittoria Monaco[1,4], Luisa Canè[2,4], Francesca Bibbò[3,4], Valentina Cioffi[5], Flora Cozzolino[1,4], Alfredo Guarino[5], Massimo Zollo[3,4], Maria Monti[1,4*]

[1]Department of Chemical Sciences, University of Naples “Federico II”, Naples, Italy

[2]Department of Translational Medical Sciences, University of Naples “Federico II”, Naples, Italy

[3]Department of Molecular Medicine and Medical Biotechnologies (DMMBM), University of Naples “Federico II”, Naples, Italy

[4]CEINGE Advanced Biotechnologies, Naples, Italy

[5]Department of Translational Medical Science, Section of Pediatrics, University of Naples “Federico II”, Naples, Italy

1. Supplementary Figures

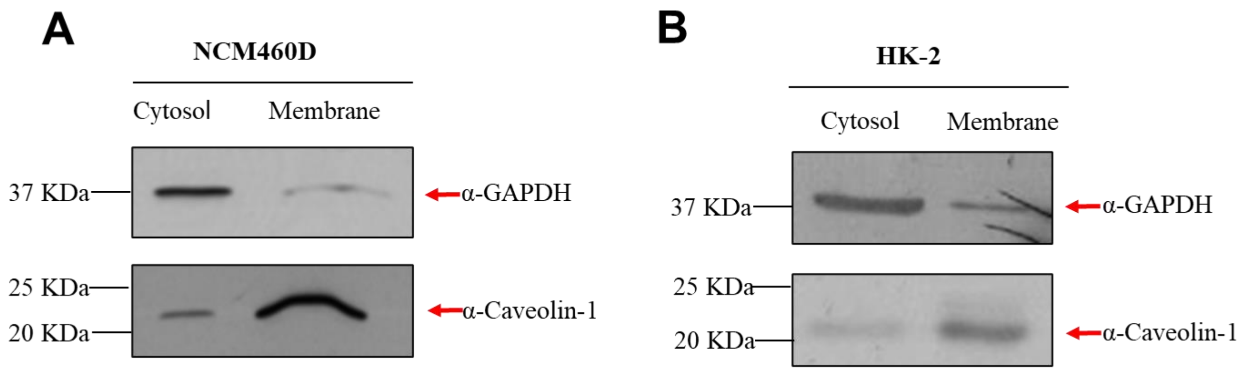

Supplementary Figure 1. Western blot assays for the verification of the fractionated lysis. For NCM460D (A panel) and HK-2 (B panel) cell lines, the presence of glyceraldehyde-3-phosphate dehydrogenase (GAPDH) and Caveolin-1 was monitored as markers of cytosolic and membrane fractions, respectively. The gel lanes loaded with the sample derived from the specific cell line, as well

as the cytosolic and membrane extracts were reported. Red arrows highlight the bands corresponding to GAPDH and Caveolin-1.

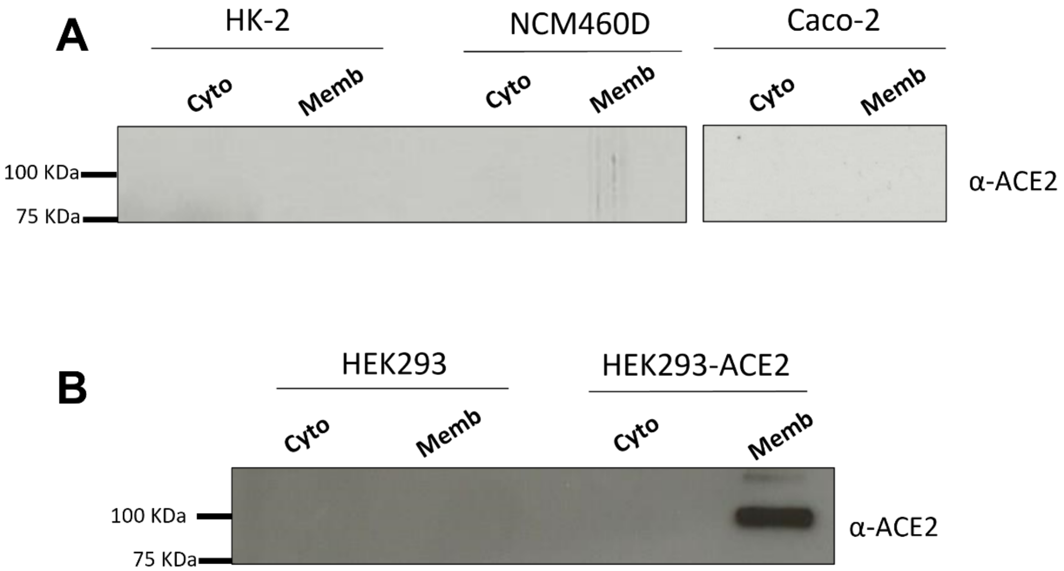

Supplementary Figure 2. Western blot assays for the assessment of ACE2 abundance. For each cell line, the cytosolic and membrane fractions are labeled as cyto and memb, respectively. In panel A: the Western Blot images for the HK-2, NCM460D, and Caco-2 cell lines are reported. In panel B: the ACE2 monitoring are reported for HEK293, HEK293-ACE2 cell lines. The latter is a cell line overexpressing ACE2, and was used as positive control.

2. Supplementary Tables

Supplementary Table 1. Details of protein identification by mass spectrometry.

Supplementary Table 2. ClueGO output based on the Biological processes ontology.

Supplementary Table 3. ClueGO output based on the Reactome pathways database.
